# *N*-acetylcysteine (NAC) in neurological disorders: mechanisms of action and therapeutic opportunities

**DOI:** 10.1002/brb3.208

**Published:** 2014-01-13

**Authors:** Reza Bavarsad Shahripour, Mark R Harrigan, Andrei V Alexandrov

**Affiliations:** 1 Department of Neurology, Comprehensive Stroke Center, University of AlabamaBirmingham, Alabama; 2 Department of Surgery, Division of Neurosurgery, University of AlabamaBirmingham, Alabama

**Keywords:** *N*-acetylcysteine, neurological disorder, treatment

## Abstract

**Background:**

There is an expanding field of research investigating the benefits of medicines with multiple mechanisms of action across neurological disorders. *N*-acetylcysteine (NAC), widely known as an antidote to acetaminophen overdose, is now emerging as treatment of vascular and nonvascular neurological disorders. NAC as a precursor to the antioxidant glutathione modulates glutamatergic, neurotrophic, and inflammatory pathways.

**Aim and discussion:**

Most NAC studies up to date have been carried out in animal models of various neurological disorders with only a few studies completed in humans. In psychiatry, NAC has been tested in over 20 clinical trials as an adjunctive treatment; however, this topic is beyond the scope of this review. Herein, we discuss NAC molecular, intracellular, and systemic effects, focusing on its potential applications in neurodegenerative diseases including spinocerebellar ataxia, Parkinson's disease, tardive dyskinesia, myoclonus epilepsy of the Unverricht–Lundbor type as well as multiple sclerosis, amyotrophic lateral sclerosis, and Alzheimer's disease.

**Conclusion:**

Finally, we review the potential applications of NAC to facilitate recovery after traumatic brain injury, cerebral ischemia, and in treatment of cerebrovascular vasospasm after subarachnoid hemorrhage.

## Introduction

Although *N*-acetylcysteine (NAC) is widely known as an antidote to acetaminophen overdose, it has multiple other uses supported by varying levels of evidence. These diverse clinical applications are linked to its ability to support the body's antioxidant and nitric oxide systems during stress, infections, toxic assault, and inflammatory conditions (Dekhuijzen 2004). Any situation resulting in a sudden or chronic overconsumption of oxygen can lead to the production reactive oxygen species (ROS). Production of ROS can occur in:

1. mitochondria,
2. inside the capillary system, and
3. an oxidative burst induced by inflammatory cells.

Approximately 2–5% of oxygen passing through the electron transport system inside the mitochondria results in superoxide. Superoxide is the most well-known of the free radicals as it is commonly produced during the natural pathway of oxidative phosphorylation (Kerksick and Willoughby 2005). ROS are produced primarily by the mitochondria as a by-product of normal cell metabolism during conversion of molecular oxygen (O_2_) to water (H_2_O). These include superoxide radical (O_2_−•), hydrogen peroxide (H_2_O_2_), and hydroxyl radical (•OH). Peroxisomes produce H_2_O_2_ during fatty acid degradation. H_2_O_2_ is mostly degraded into water by catalase, but some molecules may also escape into the cell (Ames et al. 1993). Oxidative stress occurs when there is an imbalance between oxidants and antioxidants. ROS can modify or damage DNA, proteins, and lipids in cells by oxidation and peroxidation (Beckman and Ames 1997; Lander 1997; Adler et al. 1999; Frank et al. 2000). There are several antioxidant defense mechanisms; however, both oxidants and antioxidants have a profound impact on the expression of genes. Antioxidants include vitamins C and E, and enzymes such as superoxide dismutase (SOD), catalase, and glutathione peroxidase (GSHpx) (Yu 1994) as well as endogenous thiols, or sulfhydryl containing compounds such as glutathione (GSH) and thioredoxin (Pahl and Baeuerle 1994; Sen and Packer 1996; Arrigo 1999; Davis et al. 2001). NAC is a thiol, a mucolytic agent, and a precursor of l-cysteine and reduced GSH. NAC is a source of sulfhydryl groups in cells and scavenger of free radicals as it interacts with ROS such as OH• and H_2_O_2_ (Aruoma et al. 1989).

GSH is currently one of the most studied antioxidants as it is endogenously synthesized basically in all cells. Among many, established roles for GSH are the following:

1. antioxidant defense,
2. detoxification of electrophilic xenobiotics,
3. modulation of redox (oxidation–reduction reaction)-regulated signal transduction,
4. storage and transport of cysteine,
5. regulation of cell proliferation, synthesis of deoxyribonucleotide synthesis,
6. regulation of immune responses, as well as
7. regulation of leukotriene and prostaglandin metabolism.

GSH has an important role in maintaining the redox state of the cell (Kerksick and Willoughby 2005). It thereby exerts a profound protective effect on cells. Of the three amino acids in the GSH structure (glutamate, glycine, and cysteine), cysteine has the lowest intracellular concentration (Aruoma et al. 1989). Cysteine availability can limit the rate of GSH synthesis during times of oxidative stress. NAC is an acetylated cysteine residue able to increase cell protection to oxidative stress. NAC is an effective scavenger of free radicals as well as a major contributor to maintenance of the cellular GSH status. NAC can minimize the oxidative effect of ROS through correcting or preventing GSH depletion (Kerksick and Willoughby 2005).

By doing so, NAC may decrease the inflammation that occurs in conditions such as chronic obstructive pulmonary disease (COPD), influenza, and idiopathic pulmonary fibrosis. In addition to its antioxidant action, NAC acts as a vasodilator by facilitating the production and action of nitric oxide. This property is an important mechanism of action in the prophylaxis of contrast-induced nephropathy and the potentiation of nitrate-induced vasodilation (Millea 2009).

Uses of NAC in different diseases including cancer, cardiovascular diseases, human immunodeficiency virus (HIV) infections, acetaminophen-induced liver toxicity and metal toxicity have been reviewed previously (Kelly 1998; *N*-acetylcysteine 2000). This review focuses on the recent studies on the effects of NAC in a variety of neurological disorders and brain functions in animals and humans (Table 1).

**Table 1:** Summary of NAC mechanisms of action across different neurological disorders.

Disease	Mechanism
Neurodegenerative disorders: SCD, tardive dyskinesia, myoclonus epilepsy, Unverricht–Lundbor type	Antioxidant effect by free-radical scavenging and increased levels of glutathione (Arakawa and Ito 2007)
Down syndrome	Increase and modulation of the level of super oxidase dismutase (Busciglio and Yankner 1995; Behar and Colton 2003)
Multiple sclerosis	Free-radical scavenging and inhibition of TNF toxicity (Lehmann et al. 1994; Stanislaus et al. 2005)
Amyotrophic lateral sclerosis	Increasing the level of glutathione peroxidase and free-radical scavenging (Rosen et al. 1993; Louwerse et al. 1995)
Parkinson's disease	Increasing the level of glutathione and free-radical scavenging (Schapira et al. 1990; Martínez et al. 1999)
Huntington's disease	Free-radical trapping and preventing mitochondrial dysfunction (Fontaine et al. 2000; Stanislaus et al. 2005)
Alzheimer's disease	Increasing the level of glutathione (Adair et al. 2001; Tchantchou et al. 2005; Tucker et al. 2005)
Focal cerebral ischemia	NOS inhibition, regeneration of endothelium-derived relaxing factor, increasing glutathione levels, improving microcirculatory blood flow, and tissue oxygenation (Dawson and Dawson 1997; Cuzzocrea et al. 2000b)
Subarachnoid hemorrhage	Free-radicals scavenger, endothelial apoptosis inhibition, lipid peroxidation reduction, increasing glutathione levels, and SOD enzymatic activities, endothelial integrity protection (Findlay et al. 1989; Sen et al. 2006)
Traumatic brain injury	Repair of TBI-induced mitochondrial dysfunction, increasing the reduced antioxidant enzyme and glutathione levels, inhibition of the activation of NF-*κ*B and TNF-*α* (Hoffer et al. 2002; Akca et al. 2005; Hsu et al. 2006; Chen et al. 2008)

### Basic pharmacology of NAC

**Figure 1:**
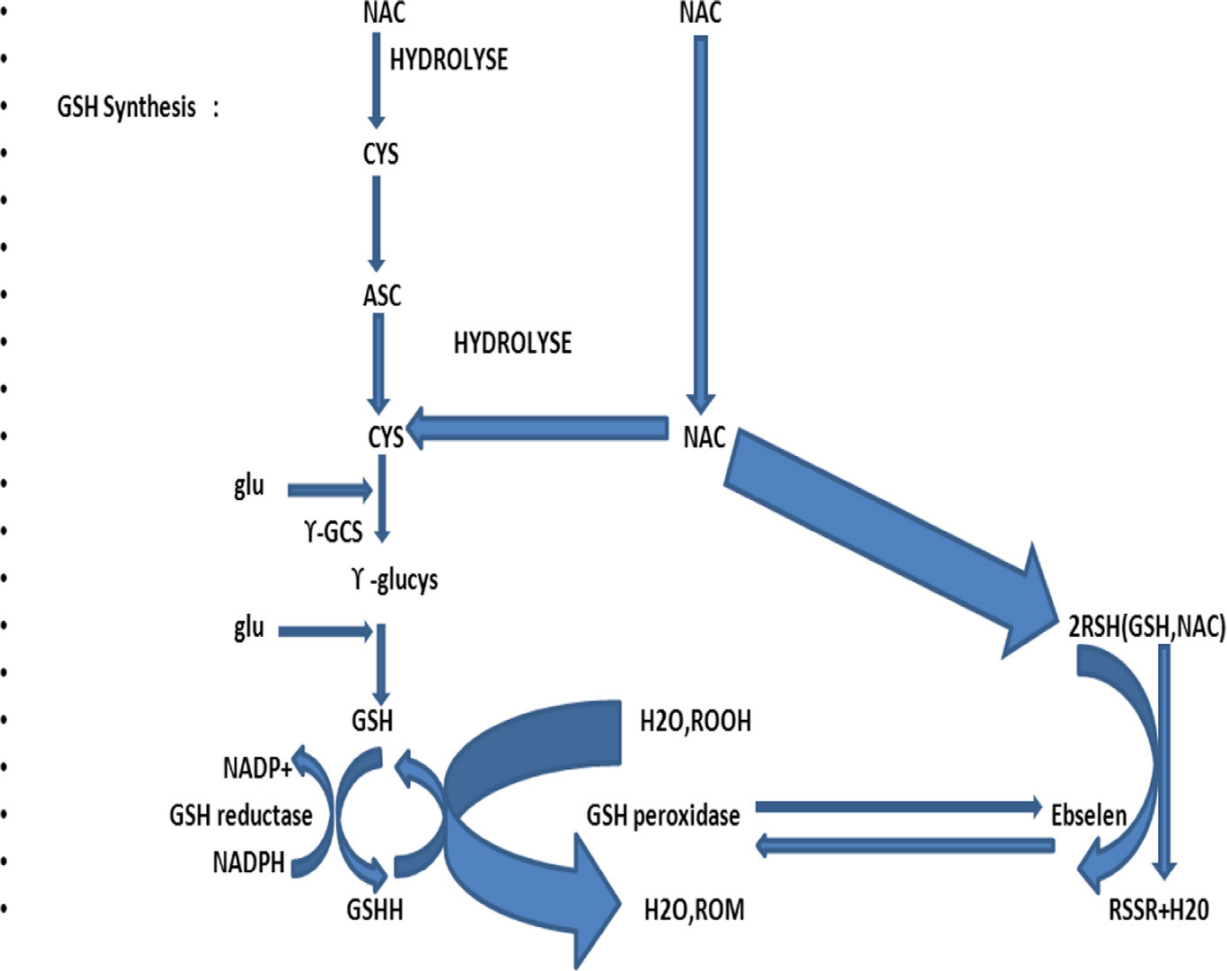
Mechanism of action of *N*-acetylcysteine (NAC). ASC, alanine-serine-cysteine (ASC) transport system; c-GCS, c-glutamylcysteine synthetase; cys, cysteine; glu, glutamine; gly, glycine; GSH, glutathione.

NAC exerts survival-promoting effects in several cellular systems (Mayer and Noble 1994). Cysteine is transported mainly by the alanine-serine-cysteine (ASC) system, a ubiquitous system of Na^+^-dependent neutral amino acid transport in a variety of cells (Bannai and Tateishi 1986; Ishige et al. 2005). NAC, however, is a membrane-permeable cysteine precursor that does not require active transport and delivers cysteine to the cell in a unique way (Fig. 1) (Sen 1997). After free NAC enters a cell, it is rapidly hydrolyzed to release cysteine, a precursor of GSH. GSH is synthesized by the coactions of c-glutamylcysteine synthetase and GSH synthetase. The synthesis of GSH is limited by the availability of substrates; cysteine is usually the limiting precursor (Meister 1995). C-glutamylcysteine synthetase is inhibited by feedback from GSH (Richman and Meister 1975). In addition, intracellular GSH is maintained in its thiol form by GSH reductase, which requires NADPH (Sen 1997). GSH participates nonenzymatically and enzymatically in protection against oxidative damage caused by ROS. GSH peroxidase catalyzes the destruction of H_2_O_2_ and hydroperoxides (Meister 1995). Thus, NAC is an antioxidant and a free-radical scavenging agent that increases intracellular GSH, a major component of the pathways by which cells are protected from oxidative stress (Arakawa and Ito 2007). Low bioavailability of NAC is one of the major limitations for maximizing its effects on oxidative stress-related diseases. Giustarini et al. (2012) reported that esterification of the carboxyl group of NAC to produce *N*-acetylcysteine ethyl ester (NACET) would increase the lipophilicity of NAC as the mechanism of increasing its pharmacokinetics. They showed that NACET is rapidly absorbed in rats after oral administration, but reaches very low concentrations in plasma. This is due to a unique feature of NACET: it rapidly enters the cells and transforms into NAC and cysteine (Giustarini et al. 2012). After oral treatment, NACET (but not NAC) was able to increase significantly the GSH content of most tissues in the rat (including brain), and protected them from paracetamol intoxication. To overcome this limitation of NAC, an amide derivative, *N*-acetylcysteine amide (NACA) has been synthesized to improve its lipophilicity, membrane permeability, and antioxidant properties. Recent studies have demonstrated the blood–brain barrier permeability and therapeutic potentials of NACA in neurological disorders (Sunitha et al. 2013).

### Does NAC cross cell membranes and the blood–brain barrier?

The ability to cross the blood–brain barrier (BBB) by a compound is thought critical to a treatment targeting dysfunction of brain parenchyma. NAC's ability to cross BBB is being disputed, and this controversy likely stems from differential ability of NAC to cross BBB that could be dependent on its dose and administration.

*N*-deacetylation and a carrier-mediated active transport are the only pathways of compound crossing the blood vessel wall. Different forms of NAC and different routes of administration may result in different concentrations and variable utilization of these mechanisms. For example, after 2 h following oral administration of S-NAC to rats, the highest concentration was seen in the kidney and liver, followed by the adrenal glands, lung, spleen, blood, muscle, and brain (Sheffner et al. 1966). Intraperitoneal or intravenous injection of 14C-NAC resulted in its uptake by most tissues except the brain and spinal cord in mice (Sunitha et al. 2013). In contrast, some studies reported that intra-arterial (intracarotid) and intravenous (jugular) administration of 14C-NAC resulted in good BBB permeability (McLellan et al. 1995).

BBB crossing by 14C-NAC increased following intraperitoneal administration of lipopolysaccharide (LPS). Interestingly, NAC-amide (NACA is an active derivation of NAC) has been measured in brain after oral or interperitoneal administration, but not NAC itself (Samuni et al. 2013). When NAC was replaced with NAC ethyl ester, a dramatic increase in the brain levels of NAC and cysteine was detected probably due to a rapid hydrolysis of NAC ethyl ester (Samuni et al. 2013).

### Effect of NAC on the functions of vascular smooth muscle cells

Excessive proliferation of vascular smooth muscle cells (VSMCs) contributes to atherosclerosis, a major cause of cerebrovascular disease. NAC partially inhibits ox-low-density lipoprotein (ox-LDL, a pro-oxidant) and urotensin-(a potent vasoconstrictor) stimulated proliferation of VSMCs. These effects of NAC raise the possibility of a therapeutic benefit to prevent stroke or atherosclerosis progression in patients with hypertension and hypercholesterolemia (*N*-acetylcysteine 2000). Additionally, NAC inhibited serum PDGF- and thrombin-stimulated extracellular single-regulated kinase (ERK2), c-JUN N-terminal kinase (JNK1), and p38 mitogen-activated protein kinase (MAPK) activation as well as expression of the c-*Fos* (70%), c-*Jun* (50%) and *JunB* (70%) genes, suggesting redox-sensitive mechanisms for protective effects of NAC in patients with major vascular risk factors (Su et al. 2001). Furthermore, NAC almost completely inhabits the Ag II-induced downregulation of AT (Dekhuijzen 2004)-R mRNA (Angiotensin II receptor, *type* 1) (Ichiki et al. 2001). NAC also blocks serotonin-stimulated superoxide production and ERK-MAPK phosphorylation in VSMCs (Lee et al. 1999). As a result of these multiple mechanisms of action, NAC reduced thickening of the neointima by 39% in rabbit aorta after injury produced by balloon (Ghigliotti et al. 2001). Finally, NAC inhibits cyclooxygenase-2 induction by benzopyrene, an atherogenic component of cigarette smoking (Yan et al. 2000).

### Role of NAC in atherosclerotic plaque stability

ROS such as superoxide, nitric oxide (NO), and H_2_O_2_ can modulate the activities of matrix-degrading proteases, matrix metalloproteinases (MMPs) and contribute to the instability of a vulnerable atherosclerotic plaque (Xu et al. 1999). Ox-LDL activates AP-1 and NF-*k*B transcription factors, promotes macrophage-mediated matrix disruption in the rupture-prone atherosclerotic plaques (Xu et al. 1999). NAC inhibits the homocysteine-enhanced expression of an ox-LDL receptor, lox-1 in the endothelium (Nagase et al. 2001). NAC can inhibit MMP-9 (gelatinase B) activity and expression in lipid-laden macrophage-derived foam cell by 60% (Galis et al. 1998) demonstrating a potential for antioxidants to stabilize vulnerable plaques. In a rabbit model, NAC reduced angioplasty-induced vascular inflammation, thrombus formation, and laminal damage (Mass et al. 1995). In hypertensive rats, NAC administration was partially protective against peroxynitrite-induced aortic vascular dysfunction related to hypertension (Cabassi et al. 2001). In a rat model with ischemic heart, NAC provided protection to ischemic and reperfusion injury in part by inhibiting adhesion molecules (Cuzzocrea et al. 2000a).

In patients with elevated remnant-like lipoprotein (RLP), adhesion molecules levels decreased after treatment with another antioxidant, *a*-tocopherol (Cabassi et al. 2001). In cultured endothelial cells, NAC decreased RLP-induced adhesion molecules by 50–70% and repaired endothelium-dependent vasorelaxation (Doi et al. 2000). A clinical trial showed that daily oral NAC administration at 1.2 mg dose increased GSH and decreased plasma vascular cell adhesion molecule-1 (VCAM-1) levels in noninsulin-dependent diabetic patients (De Mattia et al. 1998). In the previous studies, NAC supplementation significantly improved coronary and peripheral vasodilatation by enhancing the effects of NO (Andrews et al. 2001).

### Role of NAC in neural cell survival and antiapoptotic activities

Oxidative stress causes encoded cell death or apoptosis in several pathological processes such as aging, inflammation, carcinogenesis, and neurodegeneration (Chandra et al. 2000). Studies of various cell types showed NAC growth-promoting activities. NAC increases concanavalin A-induced mitogenesis and simultaneously reduces apoptosis of B-lymphocytes (Li et al. 1999; Martin et al. 2000). Interestingly, NAC and dithiothreitol (DTT) block apoptosis of endothelial cells by LPS (Abello et al. 1994).

Ox-LDL-induced superoxide production and apoptosis of human umbilical vein endothelial cells (HUVEC) were blocked by NAC (Galle et al. 1999). In contrast with endothelial cells, NAC induced apoptosis and reduced viability of rat and human VSMCs (Tsai et al. 1996). NAC was found to maintain VSMCs in inactive state, and its removal led to their return into the cell cycle (Lee et al. 1998). During investigation of the mechanisms of hyperhomocysteinemia-associated atherosclerosis, NAC suppressed homocysteine-stimulated collagen production and proliferation of VSMCs (Tyagi 1998). Such selective impact of NAC can be useful for blocking proliferation of VSMCs in atherosclerosis and lesions prone to restenosis (Yan and Greene 1998; Shirvan et al. 2000). NAC also prevented tumor necrosis factor (TNF)- and thrombin-induced neuronal cell death (Talley et al. 1995; Sarker et al. 1999). Arabinoside-induced neuron apoptosis and neurotoxicity were inhibited in vitro by NAC through ROS inhibition (Geller et al. 2001), a mechanism that supports survival of neurons. While NAC at low concentrations promotes cell growth, its high doses can lead to apoptosis (Kim et al. 2000).

### Role of NAC in cell signal cascade

The effects of NAC are most commonly attributed to its capability to scavenge ROS and elevate cellular GSH levels. However, the redox state is the principal mechanism through which ROS are integrated into cellular signal transduction pathways. As NAC affects redox-sensitive signal transduction and gene expression both in vitro and in vivo, its functions on cell signaling should also be considered.

The Rel homology domain (RHD) is a protein domain found in a family of eukaryotic transcription factors that includes a nuclear factor kappa-light-chain enhancer of activated B cells (NF-*κ*B) and a nuclear factor of activated T cells (NFAT). Some of these transcription factors appear to form multiprotein DNA-bound complexes (Wolberger 1998). NF-*κ*B represents proteins sharing RHD that bind to DNA as homo or heterodimers (p50/p65) and activate a multitude of cellular stress-related and early response genes, such as genes for cytokines, growth factors, adhesion molecules, and acute-phase proteins (Sheffner et al. 1966). NAC exerts an effect on NF-*κ*B, which has a cardinal role in regulation and expression of stress response genes under inflammatory and oxidative challenges. Interestingly, NAC affects other signal transduction pathways to expression of various genes. It can directly modulate the activity of common transcription factors both in vitro and in vivo (Samuni et al. 2013).

Oxidative stress is an effective inducer of NF-*κ*B, and NAC treatment suppressed its activation in cultured cells in vitro and in clinical sepsis also reducing subsequent cytokine production. NF-*κ*B is naturally bound to its inhibitor (I-*κ*B) that prevents its nuclear translocation. Dissociation of I-*κ*B following its phosphorylation by specific kinase of NF-*κ*B (IKK) allows NF-*κ*B transport to the nucleus. (Samuni et al. 2013).

### Misfolded proteins and neurodegenerative diseases

The alpha-helix structure of proteins is related to their function. When a protein becomes toxic, an extensive conformational change occurs and it will change to the beta-sheet (Reynaud 2010). Note that the beta-sheet conformation also exists in many functional native proteins such as the immunoglobulins. The transition from alpha-helix to beta-sheet is characteristic of amyloid deposits. Misfolded proteins appear when a protein follows the wrong folding pathway or energy-minimizing funnel, and misfolding can happen spontaneously (Reynaud 2010). As millions of copies of each protein are made during our lifetimes, sometimes a random event occurs and one of these molecules follows the wrong path, changing into a toxic configuration. This kind of conformational change is most likely to occur in proteins that have repetitive amino acid design, such as polyglutamine in Huntington's disease (HD). Under normal circumstances, proteins that have problems achieving their native configuration are helped by chaperones to fold properly. Chaperones can prevent protein misfolding by using energy (ATP). Despite chaperone actions, some proteins still misfold. Accumulation of misfolded proteins can cause disease such as amyloid diseases; Alzheimer's, Parkinson's, and HD have similar amyloid origins. Regardless of the type, the risk of getting any of these diseases increases dramatically with age (Unnithan et al. 2012). With aging or mutations, the fine balance of the synthesis, folding, and degradation of proteins will decrease resulting in the production and accumulation of misfolded proteins. Postmortem tissues from patients with neurodegenerative diseases demonstrate protein-misfolding stress and reduced proteasome activity. This broad-spectrum effect of proteotoxic stress has led to the term “proteinopathies” for neurodegenerative diseases. Unnithan and his team believe that toxic-related proteinopathies with GSH loss could have good response to NAC by reversing this GSH loss and preventing this toxicity (Unnithan et al. 2012).

### Effect of NAC on diseases of the central nervous system

Oxidative stress plays a critical role in neuronal dysfunction and death in various neurodegenerative diseases, including spinocerebellar disease (SCD), myoclonus epilepsy of the Unverricht–Lundbor type (ULD), Alzheimer's disease (AD), Parkinson's disease (PD), tardive dyskinesia (TD), and Down's syndrome (DS) (Arakawa and Ito 2007) (Table 2).

**Table 2:** Clinical trials in neurological disorders.

Trial	Status
*N*-acetylcysteine for neuroprotection in Parkinson's disease (NAC for PD)-NCT01470027	Recruiting participants
Intravenous *N*-acetylcysteine for the treatment of Gaucher's disease and Parkinson's disease. NCT01427517	Completed (Holmay et al. 2013)
*N*-acetylcysteine (NAC) for children with Tourette syndrome. NCT01172288	Recruiting participants
The role of *N*-acetyl-l-cysteine (NAC) as an adjuvant to opioid treatment in patients with chronic neuropathic pain. NCT01840345	Not yet open for participant recruitment
Single-port thoracoscopic sympathicotomy in complex regional pain syndrome type I (CRPS). NCT01886625	Not yet open for participant recruitment
A clinical trial of a vitamin/nutriceutical formulation for Alzheimer's disease. NCT01320527	Completed. Pilot Study is published (Remington et al. 2009)
NAC-003 P.L.U.S. program (Progress through Learning Understanding and Support). NCT01370954	Completed. Final results not yet published
Biomarker validation for Niemann–Pick disease, type C: safety and efficacy of *N*-acetylcysteine. NCT00975689	Completed (Fu et al. 2013)
Overcoming membrane transporters to improve CNS drug delivery—improving brain antioxidants after traumatic brain injury (Pro-NAC). NCT01322009	Recruiting participants
Efficacy mechanism of *N*-acetylcysteine in patients with posttraumatic stress disorder. NCT01664260	Not yet open for participant recruitment

#### Spinocerebellar disease

SCD is a diverse group of rare, slowly progressive neurological diseases, often inherited but of incompletely understood pathophysiology, which affect the cerebellum and its related pathways. Several studies have found evidence of oxygen-mediated damage in SCD (Arakawa and Ito 2007). If free-radical species play an important role in cerebellar degeneration in SCD, then NAC may be therapeutically effective. However, there have been no basic or clinical studies aside from one report of 18 patients with SCD who received NAC (Eldridge et al. 1983). Despite varying degrees of ataxia, dysarthria, and oculomotor disturbance among the patients, all claimed subjective improvement with NAC. The most severely affected patient was treated with NAC for 26 months, leading to a marked improvement in the eye movement control (Eldridge et al. 1983).

One case report described NAC administration in a patient with olivopontine cerebellar atrophy (OPCA) who had difficulties with balance and walking, progressive speech disruption, and diminished proprioception and pain sensitivity. A marked improvement in dysarthria and balance was seen 1 month after using NAC. By 3 months, the patient could discriminate between hot and cold, and had regained some touch and position sense (Yang et al. 1984). NAC was also administered in a case of Friedreich's ataxia, a multisystem disorder, for 8 months with an improvement in proprioception and a slight decline in ataxia (Yang et al. 1984).

Ataxia-telangiectasia (AT) is a complex multisystem disorder characterized by ataxia, ocular telangiectasia, immunodeficiency involving both T- and B-cell functions, 50- to 100-fold increased cancer incidence and increased sensitivity to ionizing radiation (Woods and Taylor 1992). Three siblings aged 7, 11, and 13 with AT, confirmed by chromosomal analysis and lymphocyte radiation fragility testing, had questionable improvement in their condition after 3 months of receiving NAC. When two patients were taken off NAC for a period of 2 weeks, rapid deterioration in their conditions ensued including a return of copious drooling in the youngest patient (Eylar et al. 1993; Sölen 1993).

#### Myoclonus epilepsy of the Unverricht–Lundbor type (PME-ULD)

PME-ULD is an autosomal recessive disorder that typically develops between the ages of 6 and 15 years with stimulus-sensitive myoclonus and tonic colonic seizures followed by progressive cerebellar syndrome (Lehesjoki and Koskiniemi 1998; Arakawa and Ito 2007). A Florida family with four siblings with PME-ULD received treatment for 20 years with phenytoin, phenobarbital, carbamazepine, and other anticonvulsants without benefit (Lehesjoki et al. 1993). After starting NAC, improvement in myoclonus was reported in the least affected patient such that she has been able to walk unaided for several days at a time. Objective measurements of improvement included some normalization of somatosensory evoked potentials (Lehesjoki et al. 1993).

#### Tardive dyskinesia

The basal ganglia are exceptionally vulnerable to free-radical overload because they are rich in dopamine as well as other catecholamines. By blocking dopamine receptors, neuroleptics may cause dopamine buildup in the basal ganglia, which then increase free-radical production. NAC decreased disease severity in both in vivo and in vitro TD models suggesting that further clinical trials may be warranted (Galili-Mosberg et al. 2000; Sadan et al. 2005).

#### Down syndrome

Down syndrome is known to involve increased systemic oxidative stress (Busciglio and Yankner 1995). The 50% overexpression of super oxidase dismutase (SOD) on chromosome 21 contributes to heightened fluxes of superoxide in all tissues. However, DS is not manifested until after birth, as the mother's antioxidant defenses might guard the fetus until delivery. Children with DS are also at significantly increased risk of Alzheimer-type dementia (Lehesjoki et al. 1993). Although NAC protects neuronal migration in DS models in vitro (Behar and Colton 2003), further clinical trials should help to clarify whether supplementation of NAC from birth can delay the beginning of Alzheimer-type dementia in DS patients.

#### Multiple sclerosis

There is a marked increase in expression of TNF in active multiple sclerosis (MS), and a correlation exists between cerebrospinal fluid levels of TNF and the severity and progression of disease (Sharief and Hentges 1991). With cytokine activation, free-radical production increases and this has been demonstrated in MS (Glabiński et al. 1993). NAC inhibits the toxicity of TNF and in an animal model of MS, it inhibited the development of MS-like pathology (Lehmann et al. 1994).

Ten patients with MS were treated with NAC for a period of up to 16 months. Due to relapsing–remitting course in many MS patients, it is difficult to determine efficacy of NAC in a small sample without concurrent controls. However, two MS patients with longstanding inability to speak coherently had a rather dramatic improvement in speech shortly after they started to take NAC. Controlled trials are needed to ascertain if NAC can decrease the number of exacerbations in MS (Stanislaus et al. 2005).

#### Huntington's disease

Mitochondrial dysfunction is a major event involved in the pathogenesis of HD. In 2000, Butterfield and his team tried to create an animal model of Huntington's disease by nitropropionic acid (3-NP) injection to rats. 3-NP is an irreversible inhibitor of complex II in the mitochondria (Fontaine et al. 2000). They reported that rats injected with 3-NP exhibited increased oxidative stress in both striatum and cortical synaptosomes. Treatment of these rats with a free-radical spin trap agent, 5-diethoxyphosphoryl-5-methyl-1-pyrroline *N*-oxide (DEPMPO) in a dose of 30 mg/kg, i.p., daily or with NAC (100 mg/kg, i.p., daily) starting 2 h before 3-NP injection protected against oxidative damage. Furthermore, both DEMPMPO and NAC treatments significantly reduced striatal lesion volumes (Fontaine et al. 2000). In 2012, Sandhir and his team evaluated the role of NAC in preventing mitochondrial dysfunction in a 3-NP-induced HD model in rat (Sandhir et al. 2012). They found an increased generation of ROS and lipid peroxidation in mitochondria of 3-NP-treated animals. Endogenous antioxidants (thiols and manganese-SOD) were decreased in mitochondria of 3-NP-treated rats. 3-NP-treated animals showed increased cytosolic cytochrome c levels and mitochondrial swelling. Increased expressions of caspase-3 and p53 were also observed in 3-NP-treated animals. Increased neural space, neurodegeneration, and gliosis accounted for most histopathologic findings in these rats. These findings were accompanied by cognitive and motor deficits. NAC treatment was capable of reversing 3-NP-induced mitochondrial dysfunction and neurobehavioral deficits in this study (Stanislaus et al. 2005), thus suggesting a beneficial effect of NAC in HD.

#### Amyotrophic lateral sclerosis

Linkage of familial amyotrophic lateral sclerosis (FALS) with mutations in the gene encoding superoxide dismutase (SOD1) support the role of free radicals in the progression of ALS (Rosen et al. 1993). Levels of SOD1 are reduced in patients with FALS, but are often normal in sporadic ALS. In two patients with sporadic ALS, SOD1 activity was normal, but GSHpx and GSH reductase activities were markedly reduced. In these patients, NAC treatment may have modified the course of the disease as one patient (duration of treatment 12 months) has remained stable with an increase in grip strength. The second patient has only marginally progressed during 17 months of treatment with NAC. Louwerse et al. (1995) reported a double-blind trial of NAC in 111 patients with ALS. Patients with limb onset but not bulbar onset of ALS had a 50% decrease in the 1 year mortality rate after beginning NAC treatment (Cray et al. 1980; May and Gray 1985; Louwerse et al. 1995).

#### Parkinson's disease

Multiple neuronal systems are involved in sporadic PD. Alpha-synuclein-immunopositive Lewy neurites and Lewy bodies are the cardinal histopathology in PD. Lesions initially occur in the dorsal motor nucleus of the glossopharyngeal and vagal nerves and anterior olfactory nucleus. Anteromedial temporal mesocortex is involved too. Neural degeneration in substantia nigra, which is a common finding in sporadic PD, could be a coincidental finding rather than a casual finding (Braak et al. 2003). Increased lipid peroxidation and dramatically decreased GSH levels have been reported in PD (Arakawa and Ito 2007). Some published reports have mentioned that GSH levels were reduced by 40% in substantia nigra compared to controls (Sian et al. 1994).

Decreased GSH levels may be due to mitochondrial dysfunction and oxidative stress. Oxidative stress will increase the accumulation of toxic forms of alpha-synuclein (SNCA). Simon and his team hypothesized that supplementation with NAC could protect against SNCA toxicity. They found that in transgenic mice, NAC increases the SN levels of GSH within 5–7 weeks of treatment; however, this increase was not sustained at 1 year. Despite this transient effect, they found that the loss of dopaminergic terminals at 1 year associated with SNCA overexpression was significantly attenuated by NAC supplementation (Clark et al. 2010).

Dopaminergic neuronal death in PD is accompanied by oxidative stress and preceded by GSH depletion. Earlier studies confirmed that mice have age-dependent loss of dopaminergic neurons in pars compacta of the substantia nigra (Jiang et al. 2005). This neuronal loss is accompanied by increased nitrotyrosine formation, nitrosylated a-synuclein, and microglial activation. These changes are considerably reduced in mice that received NAC. Martinez et al. hypothesized that treatment with a sulfur-containing antioxidant such as NAC may provide a new neuroprotective therapeutic strategy for PD (Schapira et al. 1990; Martínez et al. 1999). Generation of hydrogen peroxide by monoamino oxidase (MAO) and ROS production by catecholamines in the substantia nigra are other precipitating factors in PD (Martínez et al. 1999). Moreover, the substantia nigra in PD patients is rich in iron and in neuromelanin, two other sources that may mediate the formation of ROS. Clinical trials using antioxidants such as vitamin E (The Parkinson Study Group 1993; Pappert et al. 1996) and vitamin C for the treatment of PD have been reported (Reilly et al. 1983). However, no benefit has been found, possibly because of the poor ability of antioxidants to penetrate the BBB. On the other hand, NAC may cross the BBB (Martínez et al. 1999; Farr et al. 2003), and NAC exerts a preventive effect in a 1-methyl-4-phenyl-1,2,3,6-tetrahydropyridine (MPTP)-induced mouse model of PD (Pan et al. 2009). One ongoing randomized clinical trial to evaluate the role of NAC as a neuroprotective agent in PD is currently recruiting participants (NCT01470027, http://www.clinicaltrials.gov). Further controlled trials involving administration of NAC or GSH precursors or in combination with other antioxidants are needed (Martínez et al. 1999).

#### Alzheimer's disease

AD is a multifactorial disease. There is both direct and indirect evidence of free-radical involvement in AD. Increased levels of lipid peroxides in the temporal and cerebral cortex, and decreases in GSH in cortical areas and the hippocampus have been reported in AD (Adams et al. 1991; Jenner 1994; Lohr and Browning 1995).

Most clinical trials of antioxidants for the treatment of AD have employed either tocopherol (a class of chemical compounds which many have vitamin E activity) or selegiline (also known as l-deprenyl, an irreversible and relatively selective MAO-B inhibitor). NAC has been tested in some murine models of AD, and these studies provided supportive evidence that administration of NAC blocks oxidative damage in AD (Tchantchou et al. 2005; Tucker et al. 2005). Adair et al. (2001) administered NAC in a blinded placebo-controlled trial in patients with AD. In patients with clinically diagnosed AD, treatment with NAC failed to alter the primary outcome measures. However, the results may still support future testing of NAC in AD. First, all subjects tolerated the drug well, experiencing only minor and transient adverse effects. Second, the group taking NAC showed positive effects on some secondary outcome measures. Further testing of NAC in patients with AD may determine whether it provides more benefit than vitamin E and other antioxidants (Adair et al. 2001).

#### Beneficial effect of NAC after focal cerebral ischemia

Cerebral ischemia alters the mitochondria leading to increased ROS generation (Morris et al. 2011). Initiation of the ischemic cascade affects not only neuronal signaling but also several humoral mediators and diverse humoral pathways including opioids, NO, adenosine, bradykinin, catecholamines, heat-shock proteins, heme oxygenase, tumor necrosis factor-alpha (TNF-*α*), angiotensin, and prostaglandins (Vasdekis et al. 2013).

Neural damage following stroke is promoted by a massive release of excitatory neurotransmitters such as glutamate that acts on the *N*-methyl-d-aspartate (NMDA) receptor and other receptor subtypes (Cuzzocrea et al. 2000b). Animal studies have shown that glutamate receptor antagonists reduce neuronal damage following ischemic stroke and reduce neurotoxicity (Cuzzocrea et al. 2000b). Treatment of mice with nitric oxide synthase (NOS) inhibitors and neuronal (nNOS) gene disruption can protect ischemic brain against NMDA neurotoxicity (Dawson and Dawson 1997; Cuzzocrea et al. 2000b). Most of the toxic effects of NO appear to be a result of the reaction of NO with superoxide to form a very toxic compound peroxynitrite. Cytotoxicity of peroxynitrite is related to its roles in the initiation of lipid peroxidation, inactivation of a variety of enzymes, and depletion of GSH (Cuzzocrea et al. 2000b). Interventions to reduce the generation or the effects of peroxynitrite have showed beneficial effects in a model of cerebral ischemia as well as variety of models of inflammation and shock (Dawson and Dawson 1997). NAC's antioxidant property of being a sulphydryl donor may contribute to the regeneration of endothelium-derived relaxing factor and GSH (Aruoma et al. 1989).

Positive changes in microcirculatory blood flow and tissue oxygenation after the start of NAC treatment were documented in animals (Cuzzocrea et al. 2000b). In a Mongolian gerbil model, NAC treatment increased survival and reduced hyperactivity linked to neurodegeneration induced by cerebral ischemia and reperfusion. Histological observations of the pyramidal layer of cortex showed a reduction of neuronal loss in animals that received NAC. Generally, these results show that NAC improves brain injury induced by transient cerebral ischemia (Harrison et al. 1991). Similar results were obtained in a rat model of cerebral ischemia (Khan et al. 2004). However, no data are yet available on the use of NAC in acute ischemic stroke patients.

#### Subarachnoid hemorrhage

The pathological production of free radicals and consequent lipid peroxidation are causally related to the development of cerebral vasospasm (Sen et al. 2006). Damage in the endothelium and apoptosis of endothelial cells are also contributing to cerebral vasospasm after subarachnoid hemorrhage (SAH) (Halliwell and Gutteridge 1986; Findlay et al. 1989), while protection of endothelium from apoptosis might attenuate vasospasm (Sen et al. 2006). Intraperitoneal administration of NAC was markedly effective against cerebral vasospasm development following SAH in rabbits. NAC can significantly reduce elevated lipid peroxidation and increase the level of tissue GSH and SOD enzymatic activities. Also, NAC treatment increased the luminal area and reduced wall thickness of the basilar artery. NAC markedly reduced apoptotic index and protected the endothelial integrity (Güney et al. 2010).

Our group reported a 43-year-old woman with Hunt-Hess grade 3 SAH due to a ruptured right middle cerebral artery aneurysm that was coiled and she subsequently developed severe vasospasm. She was treated with oral NAC, 600 mg twice a day, with dramatic vasospasm resolution for 24 h, confirmed by Computed Tomography Angiography and Transcranial Doppler sonography (Friehs 2014). To our knowledge, this is the first report of NAC use and its possible effect on vasospasm in a patient with SAH. We hypothesize that NAC may be a part of the preventive therapy for vasospasm by its multiple complex action mechanisms and the subject deserves further investigation.

#### Traumatic brain injury

Several experimental studies have found that NAC plays a neuroprotective role by repairing traumatic brain injury (TBI)-induced mitochondrial dysfunction and by increasing the reduced antioxidant enzyme (Xiong et al. 1999; Hicdonmez et al. 2006; Yi et al. 2006). Previous studies focused on NAC modulating oxidative stress in the brain following TBI, but did not examine the influence of NAC on inflammation, which plays an important role in the mechanisms of secondary brain damage after TBI (Morganti-Kossmann et al. 2001). Several experimental studies have confirmed that secondary brain injury can be magnified after TBI by numerous immune mediators including interleukin-1*β* (IL-1*β*), TNF-*α*, interleukin-6 (IL-6), and intercellular adhesion molecule-1 (ICAM-1) (Merrill and Benveniste 1996; Hans et al. 1999; Rancan et al. 2001). In a rat model, cortical contusions induce a concomitant and persistent upregulation of NF-*κ*B, TNF-*α*, IL-6, and ICAM-1 (Chen et al. 2008). NAC, by increasing the amount of GSH, works as a ROS scavenger resulting in cytoprotection as it also inhibits the activation of NF-*κ*B and TNF-*α* production by LPS (Hoffer et al. 2002; Akca et al. 2005; Hsu et al. 2006). These results suggest that post-TBI NAC administration may decrease inflammatory response in the injured brain, one potential mechanism by which NAC improves secondary brain damage following TBI (Chen et al. 2008). The use of NAC in humans with TBI has not been reported.

#### NAC in psychiatric disorders

Psychiatric disorders have a multifactorial etiology that involves inflammatory pathways, glutamatergic transmission, oxidative stress, GSH metabolism, mitochondrial function, neurotrophins, apoptosis, dopamine pathway, and intracellular Ca modulation (Dean et al. 2011). As NAC plays a role in most of these pathways, its effect on psychiatric disorders has been studied more extensively in the clinical setting.

More than 20 clinical trials have employed NAC as an adjunctive treatment in various psychiatric disorders. These include methamphetamine and cannabis dependence, nicotine and cocaine addiction, pathological gambling, obsessive–compulsive disorder, trichotillomania, nail biting and skin picking, schizophrenia, bipolar disorder, autism, and AD. In most of these studies, NAC had positive effects on clinical outcomes (Gere-Paszti and Jakus 2009; Samuni et al. 2013). A detailed discussion of these results is beyond the scope of this review as it is focused on neurological disorders.

### Pharmacokinetics and side effects

With oral NAC doses of 200–400 mg, the peak plasma concentration of 0.35–4 mg/L is achieved within 1–2 h after ingestion. Information on interaction with food is lacking. The volume of distribution ranges from 0.33 to 0.47 L/kg and protein binding is significant being 50% at 4 h after the dose administration.

Intravenously infused NAC rapidly forms disulfides in plasma, which prolong the existence of the drug in plasma for up to 6 h (Sarker et al. 1999). Renal clearance has been reported at 0.190–0.211 L/h per kg; however, up to 70% of the total body clearance is nonrenal.

With oral administration, reduced NAC has a terminal half-life of 6.25 h. It is believed to be rapidly metabolized and incorporated onto proteins. After oral ingestion of 200 mg NAC, the free thiol is largely undetectable, and only low levels of oxidized NAC are detectable for several hours after administration (Cotgreave and Moldeus 1987). The data also indicate that the drug is less than 5% bioavailable from the oral formulation. Further pharmacokinetic data suggest that the drug itself does not accumulate in the body, but rather in its oxidized forms and in reduced and oxidized metabolites (Holdiness 1991; Watson and McKinney 1991).

Pharmacokinetic information is controversial regarding NAC ability to cross placenta or being excreted into breast milk. NAC in the Ames test is negative; however, animal studies on embryotoxicity are equivocal (Ziment 1988). In addition, studies in pregnant women are inadequate. Therefore, NAC should be used with caution during pregnancy, and only if clearly indicated. Its major excretory product is inorganic sulfate.

NAC is generally safe and well tolerated even at high doses. Most frequently reported side effects are nausea, vomiting, and diarrhea. Therefore, oral administration is contraindicated in persons with active peptic ulcer (Ziment 1988). Biochemical and hematological adverse effects are observed, but are not clinically relevant. Drug interactions of clinical significance have been observed with paracetamol, GSH, and anticancer agents (Holdiness 1991).

Infrequently, anaphylactic reactions due to histamine release occur and can consist of rash, pruritis, angioedema, bronchospasm, tachycardia, and changes in blood pressure. In rare circumstances, intravenous administration of NAC can lead to an allergic reaction generally in the form of rash or angioedema. In addition, as with any antioxidant nutrient, NAC at therapeutic doses (even as low as 1.2 g daily) has the potential to have pro-oxidant activity and therefore it is not recommended in the absence of a significant confirmed oxidative stress (Ziment 1988). NAC strongly potentiates the effect of nitroglycerin and related medications, and caution should be used in patients receiving these agents in whom it may cause hypotension (Atkuri et al. 2007).

## Conclusions

NAC has a broad spectrum of actions and possible applications across multiple conditions and systems. As a drug, NAC represents perhaps the ideal xenobiotic, capable of directly entering endogenous biochemical processes as a result of its own metabolism. In addition, NAC may cross the BBB. In neurological diseases, there is a potential to explore doses and duration of treatment with NAC to achieve cytoprotection.

## Conflict of Interest

None declared.

## References

[b1] (2000). N-acetylcysteine. Altern. Med. Rev.

[b2] Abello PA, Fidler SA, Buchman TG (1994). Thiol reducing agents modulate induced apoptosis in porcine endothelial cells. Shock.

[b3] Adair JC, Knoefel JE, Morgan N (2001). Controlled trial of N-acetylcysteine for patients with probable Alzheimer's disease. Neurology.

[b4] Adams JD, Klaidman LK, Odunze IN, Shen HC, Miller CA (1991). Alzheimer's and Parkinson's disease. Brain levels of glutathione, glutathione disulfide, and vitamin E. Mol. Chem. Neuropathol.

[b5] Adler V, Yin Z, Tew KD, Ronai Z (1999). Role of redox potential and reactive oxygen species in stress signaling. Oncogene.

[b6] Akca T, Canbaz H, Tataroglu C, Caglikulekci M, Tamer L, Colak T (2005). The effect of N-acetylcysteine on pulmonary lipid peroxidation and tissue damage. J. Surg. Res.

[b7] Ames BN, Shigenaga MK, Hagen TM (1993). Oxidants, antioxidants, and the degenerative diseases of aging. Proc. Natl. Acad. Sci.

[b8] Andrews NP, Prasad A, Quyyumi AA (2001). N-acetylcysteine improves coronary and peripheral vascular function. J. Am. Coll. Cardiol.

[b9] Arakawa M, Ito Y (2007). N-acetylcysteine and neurodegenerative diseases: basic and clinical pharmacology. Cerebellum.

[b10] Arrigo A-P (1999). Gene expression and the thiol redox state. Free Radical Biol. Med.

[b11] Aruoma OI, Halliwell B, Hoey BM, Butler J (1989). The antioxidant action of N-acetylcysteine: its reaction with hydrogen peroxide, hydroxyl radical, superoxide, and hypochlorous acid. Free Radical Biol. Med.

[b12] Atkuri KR, Mantovani JJ, Herzenberg LA (2007). N-Acetylcysteine–a safe antidote for cysteine/glutathione deficiency. Curr. Opin. Pharmacol.

[b13] Bannai S, Tateishi N (1986). Role of membrane transport in metabolism and function of glutathione in mammals. J. Membr. Biol.

[b14] Beckman KB, Ames BN (1997). Oxidative decay of DNA. J. Biol. Chem.

[b15] Behar TN, Colton CA (2003). Redox regulation of neuronal migration in a Down Syndrome model. Free Radical Biol. Med.

[b16] Braak H, Rub K, Del Tredici U, Jansen Steur RA, de Vos EN, Braak E (2003). Staging of brain pathology related to sporadic Parkinson's disease. Neurobiol. Aging.

[b17] Busciglio J, Yankner BA (1995). Apoptosis and increased generation of reactive oxygen species in Down's syndrome neurons in vitro. Nature.

[b18] Cabassi A, Dumont EC, Girouard H, Bouchard JF, Lamontagne M, Le Jossec D (2001). Effects of chronic N-acetylcysteine treatment on the actions of peroxynitrite on aortic vascular reactivity in hypertensive rats. J. Hypertens.

[b19] Chandra J, Samali A, Orrenius S (2000). Triggering and modulation of apoptosis by oxidative stress. Free Radical Biol. Med.

[b20] Chen G, Shi J, Hu Z, Hang C (2008). Inhibitory effect on cerebral inflammatory response following traumatic brain injury in rats: a potential neuroprotective mechanism of N-acetylcysteine. Mediators Inflamm.

[b21] Clark J, Clore EL, Zheng K, Adame A, Masliah E, Simon DK (2010). Oral N-acetyl-cysteine attenuates loss of dopaminergic terminals in alpha-synuclein overexpressing mice. PLoS ONE.

[b22] Cotgreave IA, Moldeus P (1987). Methodologies for the analysis of reduced and oxidized N-acetylcysteine in biological systems. Biopharm. Drug Dispos.

[b23] Cray PN, May PC, Mundy L, Elkins J (1980). L-Glutamate toxicity in Huntington's disease fibroblasts. Biochem. Biophys. Res. Commun.

[b24] Cuzzocrea S, Mazzon E, Costantino G, Serraino I, Caputi A, De Sarro AP (2000a). Effects of n-acetylcysteine in a rat model of ischemia and reperfusion injury. Cardiovasc. Res.

[b25] Cuzzocrea S, Mazzon E, Costantino G, Serraino I, Dugo L, Calabrò G (2000b). Beneficial effects of N-acetylcysteine on ischaemic brain injury. Br. J. Pharmacol.

[b26] Davis W, Ronai Z, Tew KD (2001). Cellular thiols and reactive oxygen species in drug-induced apoptosis. J. Pharmacol. Exp. Ther.

[b27] Dawson T, Dawson V (1997). Protection of the brain from ischemia. Cerebrovasc. Dis.

[b28] De Mattia G, Bravi M, Laurenti O, Cassone-Faldetta M, Proietti A, De Luca O (1998). Reduction of oxidative stress by oral N-acetyl-L-cysteine treatment decreases plasma soluble vascular cell adhesion molecule-1 concentrations in non-obese, non-dyslipidaemic, normotensive, patients with non-insulin-dependent diabetes. Diabetologia.

[b29] Dean O, Giorlando F, Berk M (2011). N-acetylcysteine in psychiatry: current therapeutic evidence and potential mechanisms of action. J. Psychiatry Neurosci.

[b30] Dekhuijzen P (2004). Antioxidant properties of N-acetylcysteine: their relevance in relation to chronic obstructive pulmonary disease. Eur. Respir. J.

[b31] Doi H, Kugiyama K, Oka H, Sugiyama S, Ogata N, Koide SI (2000). Remnant lipoproteins induce proatherothrombogenic molecules in endothelial cells through a redox-sensitive mechanism. Circulation.

[b32] Eldridge R, Iivanainen M, Stern R, Koerber T, Wilder BJ (1983). “Baltic” myoclonus epilepsy: hereditary disorder of childhood made worse by phenytoin. Lancet.

[b33] Eylar E, Rivera-Quinones C, Molina C, Baez I, Molina F, Mercado CM (1993). N-acetylcysteine enhances T cell functions and T cell growth in culture. Int. Immunol.

[b34] Farr SA, Poon HF, Dogrukol-Ak D, Drake J, Banks WA, Eyerman E (2003). The antioxidants *α*-lipoic acid and N-acetylcysteine reverse memory impairment and brain oxidative stress in aged SAMP8 mice. J. Neurochem.

[b35] Findlay J, Weir B, Kanamaru K, Espinosa F (1989). Arterial wall changes in cerebral vasospasm. Neurosurgery.

[b36] Fontaine MA, Geddes JW, Banks A, Butterfield DA (2000). Effect of exogenous and endogenous antioxidants on 3-nitropionic acid-inducedin vivo oxidative stress and striatal lesions. J. Neurochem.

[b37] Frank J, Pompella A, Biesalski H (2000). Histochemical visualization of oxidant stress. Free Radical Biol. Med.

[b38] Friehs GM, Bavarsad Shahripour R, Alexandrov AV, Harrigan MR (2014). Treatment of symptomatic cerebral vasospasm with N-acetylcysteine. J. NeuroImag.

[b39] Fu R, Wassif CA, Yanjanin NM, Watkins-Chow DE, Baxter LL, Incao A (2013). Efficacy of N-acetylcysteine in phenotypic suppression of mouse models of Niemann-Pick disease, type C1. Hum. Mol. Genet.

[b40] Galili-Mosberg R, Gil-Ad I, Weizman A, Melamed E, Offen D (2000). Haloperidol–induced neurotoxicity–possible implications for tardive dyskinesia. J. Neural Transm.

[b41] Galis ZS, Asanuma K, Godin D, Meng X (1998). N-acetyl-cysteine decreases the matrix-degrading capacity of macrophage-derived foam cells new target for antioxidant therapy?. Circulation.

[b42] Galle J, Heermeier K, Wanner C (1999). Atherogenic lipoproteins, oxidative stress, and cell death. Kidney Int.

[b43] Geller HM, Cheng KY, Goldsmith NK, Romero AA, Zhang AL, Morris EJ (2001). Oxidative stress mediates neuronal DNA damage and apoptosis in response to cytosine arabinoside. J. Neurochem.

[b44] Gere-Paszti E, Jakus J (2009). The effect of N-acetylcysteine on amphetaminemediateddopamine release in rat brain striatal slices by ion-pair reversed-phase high performance liquid chromatography. Biomed. Chromatogr.

[b45] Ghigliotti G, Mereto E, Eisenberg PR, Martelli A, Orsi P, Sini D (2001). N-acetyl-cysteine reduces neointimal thickening and procoagulant activity after balloon-induced injury in abdominal aortae of New Zealand white rabbits. Thromb. Haemost.

[b46] Giustarini D, Milzani A, Dalle-Donne I, Tsikas D, Rossi R (2012). N-Acetylcysteine ethyl ester (NACET): a novel lipophilic cell-permeable cysteine derivative with an unusual pharmacokinetic feature and remarkable antioxidant potential. Biochem. Pharmacol.

[b47] Glabiński A, Tawsek N, Bartosz G (1993). Increased generation of superoxide radicals in the blood of MS patients. Acta Neurol. Scand.

[b48] Güney O, Erdi F, Esen H, Kiyici A, Kocaogullar Y (2010). N-acetylcysteine prevents vasospasm after subarachnoid hemorrhage. World Neurosurg.

[b49] Halliwell B, Gutteridge JM (1986). Oxygen free radicals and iron in relation to biology and medicine: some problems and concepts. Arch. Biochem. Biophys.

[b50] Hans VH, Kossmann T, Lenzlinger PM, Probstmeier R, Imhof H-G, Trentz O (1999). Experimental axonal injury triggers interleukin-6 mRNA, protein synthesis and release into cerebrospinal fluid. J. Cereb. Blood Flow Metab.

[b51] Harrison PM, Wendon JA, Gimson AE, Alexander GJ, Williams R (1991). Improvement by acetylcysteine of hemodynamics and oxygen transport in fulminant hepatic failure. N. Engl. J. Med.

[b52] Hicdonmez T, Kanter M, Tiryaki M, Parsak T, Cobanoglu S (2006). Neuroprotective effects of N-acetylcysteine on experimental closed head trauma in rats. Neurochem. Res.

[b53] Hoffer E, Baum Y, Nahir AM (2002). N-Acetylcysteine enhances the action of anti-inflammatory drugs as suppressors of prostaglandin production in monocytes. Mediators Inflamm.

[b54] Holdiness MR (1991). Clinical pharmacokinetics of N-acetylcysteine. Clin. Pharmacokinet.

[b55] Holmay MJ, Terpstra M, Coles LD, Mishra U, Ahlskog M, Oz G (2013). N-acetylcysteine boosts brain and blood glutathione in gaucher and Parkinson diseases. Clin. Neuropharmacol.

[b56] Hsu BG, Lee RP, Yang FL, Harn HJ, Chen HI (2006). Post-treatment with N-acetylcysteine ameliorates endotoxin shock-induced organ damage in conscious rats. Life Sci.

[b57] Ichiki T, Takeda K, Tokunou T, Funakoshi Y, Ito K, Iino N (2001). Reactive oxygen species-mediated homologous downregulation of angiotensin II type 1 receptor mRNA by angiotensin II. Hypertension.

[b58] Ishige K, Tanaka M, Arakawa M, Saito H, Ito Y (2005). Distinct nuclear factor-kappaB/Rel proteins have opposing modulatory effects in glutamate-induced cell death in HT22 cells. Neurochem. Int.

[b59] Jenner P (1994). Oxidative damage in neurodegenerative disease. Lancet.

[b60] Jiang CT, Wan XH, He Y, Pan TH, Jankovic J, Le WD (2005). Age-dependent dopaminergic dysfunction in Nurr1 knockout mice. Exp. Neurol.

[b61] Kelly GS (1998). Clinical applications of N-acetylcysteine. Altern. Med. Rev.

[b62] Kerksick C, Willoughby D (2005). The antioxidant role of glutathione and N-acetyl-cysteine supplements and exercise-induced oxidative stress. J. Int. Soc. Sports Nutr.

[b63] Khan M, Sekhon B, Jatana M, Giri S, Gilg AG, Sekhon C (2004). Administration of N-acetylcysteine after focal cerebral ischemia protects brain and reduces inflammation in a rat model of experimental stroke. J. Neurosci. Res.

[b64] Kim YH, Takahashi M, Suzuki E, Niki E (2000). Apoptosis induced by hydrogen peroxide under serum deprivation and its inhibition by antisense c-*jun* in F-MEL cells. Biochem. Biophys. Res. Commun.

[b65] Lander HM (1997). An essential role for free radicals and derived species in signal transduction. FASEB J.

[b66] Lee JS, Kypreos KE, Sonenshein GE (1998). Synchronization of cultured vascular smooth muscle cells following reversal of quiescence induced by treatment with the antioxidant N-acetylcysteine. Exp. Cell Res.

[b67] Lee S-L, Wang W-W, Finlay GA, Fanburg BL (1999). Serotonin stimulates mitogen-activated protein kinase activity through the formation of superoxide anion. Am. J. Physiol.

[b68] Lehesjoki AE, Koskiniemi M (1998). Clinical features and genetics of progressive myoclonus epilepsy of the Univerricht–Lundborg type. Ann. Med.

[b69] Lehesjoki AE, Eldridge R, Eldridge J, Wilder BJ, de la Chapelle A (1993). Progressive myoclonus epilepsy of Unverricht-Lundborg type: a clinical and molecular genetic study of a family from the United States with four affected sibs. Neurology.

[b70] Lehmann D, Karussis D, Misrachi-Koll R, Shezen E, Ovadia H, Abramsky O (1994). Oral administration of the oxidant-scavenger N-acetyl-L-cysteine inhibits acute experimental autoimmune encephalomyelitis. J. Neuroimmunol.

[b71] Li AE, Ito H, Rovira II, Kim K-S, Takeda K, Yu Z-Y (1999). A role for reactive oxygen species in endothelial cell anoikis. Circ. Res.

[b72] Lohr JB, Browning JA (1995). Free radical involvement in neuropsychiatric illness. Psychopharmacol. Bull.

[b73] Louwerse ES, Weverling GJ, Bossuyt PM, Meyjes F, de Jong J (1995). Randomized, double-blind, controlled trial of acetylcysteine in amyotrophic lateral sclerosis. Arch. Neurol.

[b74] Martin KR, Kari FW, Barrett JC, French JE (2000). N-acetyl-L-cysteine simultaneously increases mitogenesis and suppresses apoptosis in mitogen-stimulated B-lymphocytes from p53 haploinsufficient Tg. AC (v-Ha-ras) mice. In Vitr. Mol. Toxicol.

[b75] Martínez M, Martínez N, Hernández AI, Ferrándiz ML (1999). Hypothesis: can N-acetylcysteine be beneficial in Parkinson's disease?. Life Sci.

[b76] Mass H, Pirazzi B, Gonzalez P, Collazo V, Fitzovich D, Avakian E (1995). N-acetylcysteine diminishes injury induced by balloon angioplasty of the carotid artery in rabbits. Biochem. Biophys. Res. Commun.

[b77] May PC, Gray PN (1985). The mechanism of glutamate-induced degeneration of cultured Huntington's disease and control fibroblasts. J. Neurol. Sci.

[b78] Mayer M, Noble M (1994). N-acetyl-L-cysteine is a pluripotent protector against cell death and enhancer of trophic factor-mediated cell survival in vitro. Proc. Natl. Acad. Sci.

[b79] McLellan LI, Lewis AD, Hall DJ, Ansell JD, Wolf CR (1995). Uptake and distribution of N-acetylcysteine in mice: tissue-specific effects on glutathione concentrations. Carcinogenesis.

[b80] Meister A (1995). Glutathione metabolism. Methods Enzymol.

[b81] Merrill JE, Benveniste EN (1996). Cytokines in inflammatory brain lesions: helpful and harmful. Trends Neurosci.

[b82] Millea PJ (2009). N-acetylcysteine: multiple clinical applications. Am. Fam. Physician.

[b83] Morganti-Kossmann MC, Rancan M, Otto VI, Stahel PF, Kossmann T (2001). Role of cerebral inflammation after traumatic brain injury: a revisited concept. Shock.

[b84] Morris KC, Lin HW, Thompson JW, Perez-Pinzon MA (2011). Pathways for ischemic cytoprotection: role of sirtuins in caloric restriction, resveratrol, and ischemic preconditioning. J. Cereb. Blood Flow Metab.

[b85] Nagase M, Ando K, Nagase T, Kaname S, Sawamura T, Fujita T (2001). Redox-sensitive regulation of lox-1 gene expression in vascular endothelium. Biochem. Biophys. Res. Commun.

[b86] Pahl HL, Baeuerle PA (1994). Oxygen and the control of gene expression. BioEssays.

[b87] Pan J, Xiao Q, Sheng CY, Hong Z, Yang HQ, Wang G (2009). Blockade of the translocation and activation of c-Jun N-terminal kinase 3 (JNK3) attenuates dopaminergic neuronal damage in mouse model of Parkinson's disease. Neurochem. Int.

[b88] Pappert E, Tangney C, Goetz C, Ling Z, Lipton J, Stebbins G (1996). Alpha-tocopherol in the ventricular cerebrospinal fluid of Parkinson's disease patients Dose-response study and correlations with plasma levels. Neurology.

[b89] Rancan M, Otto VI, Hans VH, Gerlach I, Jork R, Trentz O (2001). Upregulation of ICAM-1 and MCP-1 but not of MIP-2 and sensorimotor deficit in response to traumatic axonal injury in rats. J. Neurosci. Res.

[b90] Reilly D, Hershey L, Rivera-Calimlim L, Shoulson I (1983). On-off effects in Parkinson's disease: a controlled investigation of ascorbic acid therapy. Adv. Neurol.

[b91] Remington R, Chan A, Paskavitz J, Shea TB (2009). Efficacy of a vitamin/nutriceutical formulation for moderate-stage to later-stage Alzheimer's disease: a placebo-controlled pilot study. Am. J. Alzheimers Dis. Other Demen.

[b92] Reynaud E (2010). Protein misfolding and degenerative diseases. Nat. Educ.

[b93] Richman PG, Meister A (1975). Regulation of gamma-glutamyl-cysteine synthetase by nonallosteric feedback inhibition by glutathione. J. Biol. Chem.

[b94] Rosen DR, Siddique T, Patterson D, Figlewicz DA, Sapp P, Hentati A (1993). Mutations in Cu/Zn superoxide dismutase gene are associated with familial amyotrophic lateral sclerosis. Nature.

[b95] Sadan O, Bahat-Stromza M, Gilgun-Sherki Y, Atlas D, Melamed E, Offen D (2005). A novel brain-targeted antioxidant (AD4) attenuates haloperidol-induced abnormal movement in rats: implications for tardive dyskinesia. Clin. Neuropharmacol.

[b96] Samuni Y, Goldstein S, Dean OM, Berk M (2013). The chemistry and biological activities of N-acetylcysteine. Biochim. Biophys. Acta.

[b97] Sandhir R, Sood A, Mehrotra A, Kamboj SS (2012). N-Acetylcysteine reverses mitochondrial dysfunctions and behavioral abnormalities in 3-nitropropionic acid-induced Huntington's disease. Neurodegener. Dis.

[b98] Sarker KP, Abeyama K, Nishi J, Nakata M, Tokioka T, Nakajima T (1999). Inhibition of thrombin-induced neuronal cell death by recombinant thrombomodulin and E5510, a synthetic thrombin receptor signaling inhibitor. Thromb. Haemost.

[b99] Schapira A, Cooper J, Dexter D, Clark J, Jenner P, Marsden C (1990). Mitochondrial complex I deficiency in Parkinson's disease. J. Neurochem.

[b100] Sen CK (1997). Nutritional biochemistry of cellular glutathione. J. Nutr. Biochem.

[b101] Sen CK, Packer L (1996). Antioxidant and redox regulation of gene transcription. FASEB J.

[b102] Sen O, Caner H, Aydin MV, Ozen O, Atalay B, Altinors N (2006). The effect of mexiletine on the level of lipid peroxidation and apoptosis of endothelium following experimental subarachnoid hemorrhage. Neurol. Res.

[b103] Sharief MK, Hentges R (1991). Association between tumor necrosis factor-*α* and disease progression in patients with multiple sclerosis. N. Engl. J. Med.

[b104] Sheffner AL, Medler EM, Bailey KR, Gallo DG, Mueller AG, Sarett HP (1966). Metabolic studies with acetylcysteine. Biochem. Pharmacol.

[b105] Shirvan A, Shina R, Ziv I, Melamed E, Barzilai A (2000). Induction of neuronal apoptosis by Semaphorin3A-derived peptide. Mol. Brain Res.

[b106] Sian J, Dexter DT, Lees AJ, Daniel S, Agid Y, Javoy-Agid F (1994). Alterations in glutathione levels in Parkinson's disease and other neurodegenerative disorders affecting basal ganglia. Ann. Neurol.

[b107] Sölen G (1993). Radioprotective effect of N-acetylcysteine in vitro using the induction of DNA breaks as end-point. Int. J. Radiat. Biol.

[b108] Stanislaus R, Gilg AG, Singh AK, Singh I (2005). N-acetyl-L-cysteine ameliorates the inflammatory disease process in experimental autoimmune encephalomyelitis in Lewis rats. J. Autoimmune Dis.

[b109] Su B, Mitra S, Gregg H, Flavahan S, Chotani MA, Clark KR (2001). Redox regulation of vascular smooth muscle cell differentiation. Circ. Res.

[b110] Sunitha K, Hemshekhar M, Thushara RM, Santhosh MS, Yariswamy M, Kemparaju K (2013). N-Acetylcysteine amide: a derivative to fulfill the promises of N-Acetyl cysteine. Free Radic. Res.

[b111] Talley AK, Dewhurst S, Perry SW, Dollard SC, Gummuluru S, Fine SM (1995). Tumor necrosis factor alpha-induced apoptosis in human neuronal cells: protection by the antioxidant N-acetylcysteine and the genes bcl-2 and crmA. Mol. Cell. Biol.

[b112] Tchantchou F, Graves M, Rogers E, Ortiz D, Shea TB (2005). N-acteyl cysteine alleviates oxidative damage to central nervous system of ApoE-deficient mice following folate and vitamin E-deficiency. J. Alzheimers Dis.

[b113] The Parkinson Study Group (1993). Effects of tocopherol and deprenyl on the progression of disability in early Parkinson's disease. N. Engl. J. Med.

[b114] Tsai J-C, Jain M, Hsieh C-M, Lee W-S, Yoshizumi M, Patterson C (1996). Induction of apoptosis by pyrrolidinedithiocarbamate and N-acetylcysteine in vascular smooth muscle cells. J. Biol. Chem.

[b115] Tucker S, Ahl M, Bush A, Westaway D, Huang X, Rogers JT (2005). Pilot study of the reducing effect on amyloidosis in vivo by three FDA pre-approved drugs via the Alzheimer's APP 5′ untranslated region. Curr. Alzheimer Res.

[b116] Tyagi SC (1998). Homocysteine redox receptor and regulation of extracellular matrix components in vascular cells. Am. J. Physiol.

[b117] Unnithan AS, Choi HJ, Titler AM, Posimo JM, Leak RK (2012). Rescue from a two hit, high-throughput model of neurodegeneration with N-acetyl cysteine. Neurochem. Int.

[b118] Vasdekis SN, Athanasiadis D, Lazaris A, Martikos G, Katsanos AH, Tsivgoulis G (2013). The role of remote ischemic preconditioning in the treatment of atherosclerotic diseases. Brain Behav.

[b119] Watson WA, McKinney PE (1991). Activated charcoal and acetylcysteine absorption: issues in interpreting pharmacokinetic data. DICP.

[b120] Wolberger C (1998). Combinatorial transcription factors. Curr. Opin. Genet. Dev.

[b121] Woods C, Taylor A (1992). Ataxia telangiectasia in the British Isles: the clinical and laboratory features of 70 affected individuals. Q. J. Med.

[b122] Xiong Y, Peterson P, Lee C (1999). Effect of N-acetylcysteine on mitochondrial function following traumatic brain injury in rats. J. Neurotrauma.

[b123] Xu X-P, Meisel SR, Ong JM, Kaul S, Cercek B, Rajavashisth TB (1999). Oxidized low-density lipoprotein regulates matrix metalloproteinase-9 and its tissue inhibitor in human monocyte-derived macrophages. Circulation.

[b124] Yan CYI, Greene LA (1998). Prevention of PC12 cell death by N-acetylcysteine requires activation of the Ras pathway. J. Neurosci.

[b125] Yan Z, Subbaramaiah K, Camilli T, Zhang F, Tanabe T, McCaffrey TA (2000). Benzo[a]pyrene induces the transcription of cyclooxygenase-2 in vascular smooth muscle cells. Evidence for the involvement of extracellular signal-regulated kinase and NF-kappaB. J. Biol. Chem.

[b126] Yang GQ, Chen JS, Wen ZM, Ge KY, Zhu LZ, Chen XC (1984). The role of selenium in Keshan disease. Adv. Nutr. Res.

[b127] Yi J-H, Hoover R, McIntosh TK, Hazell AS (2006). Early, transient increase in complexin I and complexin II in the cerebral cortex following traumatic brain injury is attenuated by N-acetylcysteine. J. Neurotrauma.

[b128] Yu B (1994). Cellular defenses against damage from reactive oxygen species. Physiol. Rev.

[b129] Ziment I (1988). Acetylcysteine: a drug that is much more than a mucokinetic. Pharmacother.

